# Exploring the Association Between Third Molar Agenesis and Carabelli Traits: A Cross-Sectional Study

**DOI:** 10.3390/dj13010023

**Published:** 2025-01-06

**Authors:** Isabela Ribeiro Madalena, Heloisa Guimarães Resende, Ariane Beatriz Blancato, Maria Angélica Hueb de Menezes-Oliveira, Flares Baratto-Filho, Poliana Ferreira Santos, Camila Paiva Perin, Thais Vilalba Paniagua Machado do Nascimento, Peter Proff, Christian Kirschneck, César Penazzo Lepri, Erika Calvano Küchler

**Affiliations:** 1 Department of Biomaterials, University of Uberaba, Uberaba 38010-200, Brazil; isabelarmadalena@hotmail.com (I.R.M.); heloisag.resende@gmail.com (H.G.R.); angelicahueb@hotmail.com (M.A.H.d.M.-O.); cesarlepri@yahoo.com.br (C.P.L.); 2 School of Dentistry, Presidente Tancredo de Almeida Neves University Center, São João del Rei 36307-251, Brazil; poliana.santos@uniptan.edu.br; 3 School of Dentistry, University of Tuiuti of Paraná, Curitiba 82010-330, Brazil; fbaratto1@gmail.com; 4 School of Dentistry, University of the Joinville Region, Joinville 89219-710, Brazil; camila.perin@utp.br (C.P.P.); thaisvilalba@gmail.com (T.V.P.M.d.N.); 5 Department of Orthodontics, University of Regensburg, 93053 Regensburg, Germany; peter.proff@ukr.de; 6 Department of Orthodontics, University Hospital Bonn, Medical Faculty, University of Bonn, 53111 Bonn, Germany; christian.kirschneck@uni-bonn.de

**Keywords:** Carabelli trait, dental agenesis, molar, third, tooth abnormalities

## Abstract

**Background/Objectives:** Dental agenesis is the congenital absence of at least one tooth and has been associated with several other developmental dental conditions, such as morphological dental alterations and Carabelli trait. This study sought to investigate whether third molar agenesis is associated with Carabelli traits in permanent molars. **Methods**: This was a cross-sectional study that used a convenience sample obtained from the orthodontic records of German patients. Patients with syndromes, oral clefts, congenital alterations including dental agenesis (except agenesis of third molars), and severe cases of bruxism with a loss of tooth tissue were excluded. Teeth with cavitated lesions of dental caries, occlusal wear, restorations, and evident dental deformities were not included in the evaluation. The Carabelli trait was evaluated in the permanent maxillary molars. The Carabelli trait was classified according to its expression for each tooth as either absent, negative, and positive expressions. Third molar agenesis was evaluated only in patients older than 10 years old (based on when initial tooth formation should be visible in the panoramic radiographs). The two-sided Chi-squared test was used to investigate the association between the conditions, using an alpha of 5% (*p* < 0.05). **Results**: A total of 155 patients (74 females and 81 males) were investigated; 39 had third molar agenesis and 75 had the Carabelli trait. There was no statistical significance difference between patients with third molar agenesis and those with Carabelli traits in relation to sex (*p* > 0.05). The Carabelli trait was more common in first molars than in second molars. There was no statistical significant association between third molar agenesis and Carabelli traits (*p* > 0.05). **Conclusions**: Third molar agenesis was not associated with the Carabelli trait in the permanent molars in this population.

## 1. Introduction

Dental agenesis, also known as tooth agenesis, is a common craniofacial developmental anomaly characterized by the congenital absence of one or more dental elements. It is one of the most common developmental dental anomalies and can affect both primary and permanent dentition [1,2,3]. Tooth agenesis can vary in severity, ranging from the absence of a single dental element to the total failure of teeth formation. The congenital absence of one tooth to five teeth is called hypodontia. Severe cases are called oligodontia and are characterized by the absence of six or more teeth (excluding third molars). The complete failure of tooth development is known as anodontia. Anadontia is very rare and is usually associated with genetic syndromes [2,3].

Scientific evidence suggests that dental agenesis has a multifactorial etiological nature. Genetic and non-genetic factors can be involved in the etiology of dental agenesis. Although dental agenesis is sporadically caused by environmental factors, such as infections, dento-facial trauma during odontogenesis, radiotherapy, or chemotherapy, the large majority of dental agenesis cases are caused by genetic factors, in which several genes which play an important role in odontogenesis could be involved in the etiology of this condition [1,2,3,4,5]. The prevalence of dental agenesis is high in several populations [6,7,8]. Some systematic reviews with meta-analysis point to an average worldwide prevalence of dental agenesis of up to 22.63% [9,10]. Studies also suggest that there is a significant difference in the frequency of dental agenesis according to demographic data, indicating a strong influence of population phenotypic characteristics [9,10]. The most commonly affected type of tooth is the third molar. Third molars, commonly known as wisdom teeth, are the last teeth to develop in humans and are the last set of molars that typically emerge in the oral cavity of humans. They are the third and final set of permanent molars to develop and erupt in humans [8].

Dental agenesis can either be an isolated phenotypic condition (non-syndromic form) or can be a part of a genetic syndrome (syndromic form), demonstrating the genetically and phenotypically heterogeneity of this complex developmental condition. Non-syndromic dental agenesis has been associated with several other developmental dental conditions, including dental morphological alterations such as molar size and shape variations. For example, dental agenesis is commonly associated with lateral maxillary incisor microdontia, peg-shaped maxillary lateral incisors, and taurodontism in permanent molars [11,12,13,14,15,16,17]. The Carabelli trait (also known as the Cusp of Carabelli) is a morphological alteration of the dental crown. Carabelli traits are accessories to dental feature, which was first designated in the 19th century by a prominent Hungarian dentist and professor of dental surgery in Vienna, named Georg Carabelli. He described it as a ‘tuberculus anomalus’, that originates from the lingual cingulum area on the mesio-lingual region of the protocone maxillary molars crowns. It is a tubercle or it is the extra fifth cusp [18,19,20]. The Carabelli trait is a characteristic morphological trait often observed on the mesiopalatal surface of the second maxillary permanent molars and deciduous second maxillary molars [18,19,20]. The prevalence of the Carabelli trait has been widely described in some populations, appearing with frequencies of from approximately 72% in deciduous dentition and 59% in permanent dentition [19].

An interesting recent study published by Kerekes-Máthé et al. (2023) have reported that the Carabelli trait was observed in significantly greater numbers of patients with the dental agenesis of lateral incisors than in non-tooth agenesis patients [17]. Third molars are the most commonly affected teeth by agenesis, occurring in approximately 20–30% of the general population and affecting up to one to four teeth [21]. Third molar agenesis has been suggested in association with variations in number, tooth position, and morphological changes [12,14], and it is possible that the Carabelli trait is also more common in patients with third molars agenesis than in patients with no agenesis. To investigate the association between different conditions could provide insights into the broader genetic basis of dentofacial development. Therefore, the present study aims to investigate whether third molar agenesis is associated with Carabelli traits in permanent molars.

## 2. Materials and Methods

### 2.1. Ethical Aspects

The ethics committee of the University of Regensburg, Germany, approved the samples collection and experiment (approval code: #12-170-0150, 13 November 2019). Informed consent/assent was obtained from all participants and/or their legal guardian. Good clinical practice guidelines and the ethical standards of the Declaration of Helsinki were followed for the present study.

### 2.2. Sample Characterization

This was a cross-sectional retrospective study that used a convenience sample obtained from the orthodontic records of German patients during dental treatment at the Orthodontics Clinic of the University of Regensburg and private orthodontic practices in Regensburg between 2020 and 2021.

This current study was carried out according to the Strengthening of the Reporting of Observational studies in Epidemiology (STROBE) checklist, which determined the quality of reporting in articles from a variety of different aspects (such as abstracts, key-words, introduction, aim, methods, results, discussion, and other important information) [22].

### 2.3. Inclusion and Exclusion Criteria

Orthodontic treatment records including dental cast and radiographs (panoramic and cephalometric) of the patients of both sexes were included. Orthodontic records with the absence of maxillary dental casts and/or the absence of panoramic radiographs were excluded from this study. Patients with underling syndromes, cleft lip and or cleft palate, congenital changes including dental agenesis of other teeth (except agenesis of third molars) and severe cases of bruxism (gridding) with loss of tooth tissue were also excluded. Teeth presenting cavitated lesions of dental caries, occlusal wear, dental crown restorations, and obvious morphological dental deformities were not included in the evaluation.

Only German patients older than 12 years old were included, which is the age when the congenital absence of the third molar could be confirmed.

### 2.4. Phenotypes Definition—Carabelli Traits

Digitalized dental casts were used to investigate the Carabelli trait in the first and in the second permanent upper molars (from left and right sides). The Arizona State University Dental Anthropology System (ASUDAS) was used to classify the Carabelli trait [23]. ASUDAS is a standardized classification system used in dental anthropology to study and compare human tooth morphology across populations. It provides a method to describe, score, and analyze variations in the shape and size of dental traits in both ancient and modern human populations. ASUDAS was developed by researchers at Arizona State University and has become a widely accepted tool in bio-archaeology, forensic anthropology, and evolutionary biology.

The classification system used the following gradations: 0—smooth mesio-buccal surface of the crown. 1—minor vertical ridge and groove. 2—minor pit with small grooves deviating from the depression. 3—double vertical edges or slight, and partial cusp outline; 4—Y-form (moderate grooves curving occlusally). 5—small tubercle. 6—extensive cusp with a moderate tubercle. 7—big tubercle with a free apex. In the classification of Dahlberg [24], grades 1 to 4 are considered negative trait forms, while grades 5 to 7 represent positive trait forms. The sample was then categorized based on the expression of the Carabelli trait into absent form, negative form, and positive form, with negative referring to a pit or groove and positive indicating a distinct cusp form.

### 2.5. Phenotypes Definition—Third Molars Agenesis

Third molar agenesis was evaluated in digital panoramic radiographs. Panoramic radiographs (or orthopantomographs) are well suited for diagnosing dental agenesis due to their ability to provide a comprehensive, wide-field view of the entire dental structure in a single image. A panoramic radiograph captures all third molars, both erupted and unerupted, or still in the developmental process, as well as the supporting structures in the maxilla and mandible. This wide scope allows the visualization of every tooth at once, making it easier to spot missing teeth due to agenesis and their patterns across both dental arches. This is particularly useful for third molar agenesis, which can affect multiple teeth in different regions of the mouth.

Third molar agenesis was defined based on the age of patients and when initial dental germ formation should be visible in the radiograph images (12 years old). The digital dental radiographs were investigated on a standard monitor under low ambient light, the contrast and brightness settings of the monitor display were also adjusted. If dental agenesis could not be confirmed, the patient’s record was excluded from the study [16]. The panoramic radiographs were only evaluated by one senior dentist, a specialist in dental agenesis (ECK). For intra-examiner reliability, we randomly chose 10% of the sample and the investigation was conducted twice in a two-week interval. The Kappa statistics showed perfect agreement (Kappa value for all third molar agenesis evaluation was 1). Patients with at least one third molar congenitally absent were included in the third molar agenesis group.

**Figure 1:**
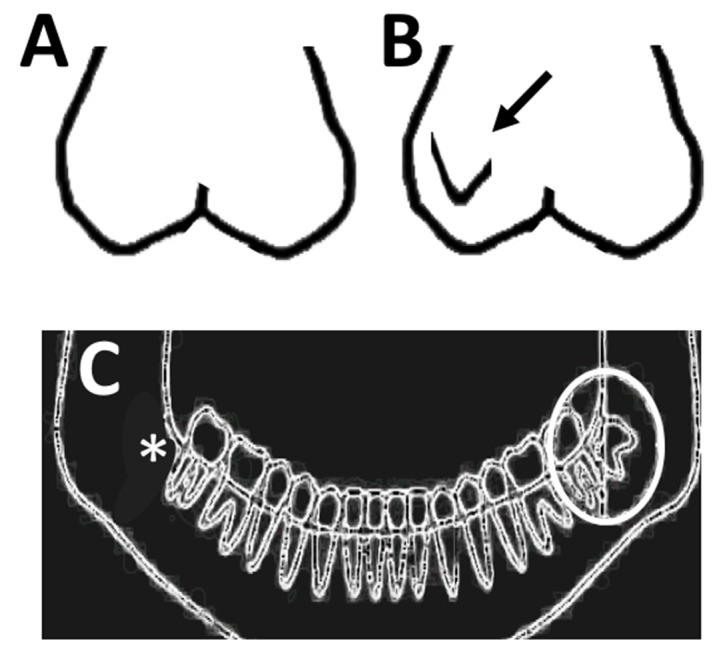
Traits definition. (**A**)— An example of a molar without the Carabelli trait. (**B**)—An example of a Carabelli trait in the molar (arrow shows the Carabelli cusp). (**C**)—A digital panoramic radiograph with a unilateral mandibular third molar agenesis (* indicated the agenesis).

Figure 1 shows an example of the Carabelli trait in first maxillary permanent molar and a molar with no Carabelli trait in the digitalized dental casts. Figure 1 also shows an example of unilateral mandibular third molar agenesis in a panoramic radiograph.

### 2.6. Statistical Analysis

The data were first recorded using Excel spreadsheets (Excel v16.16.1, Microsoft, Redmond, WA, USA). The descriptive analysis was performed and the data are presented as absolute numbers (n) and relative frequencies (%). Data were subsequently processed and analyzed with the Prism Grapdpad 9. The two-sided chi-square test was used for inferential statistics and allowed the comparison of the association between independent variables and dependent categorical variables. The frequency distribution of Carabelli traits among the third molar agenesis and non-agenesis groups were then evaluated. The analysis was also performed according to the genders. The statistical significance level was set as an alpha of 5% (*p* < 0.05) for all the evaluated comparisons.

## 3. Results

A total of 155 patients were included in the study. Of these, 74 (47.74%) were female, and 81 (52.25%) were male. Thirty-nine (25.16%) patients had third molar agenesis in at least one third molar, and 75 (48.38%) patients exhibited the Carabelli trait in at least one permanent maxillary molar.

Among the patients with third molar agenesis, 19 (48.72%) were females and 20 (51.28%) were males. Among those with the Carabelli trait, 34 (45.33%) were females and 41 (54.67%) were males. There was no statistically significant difference between patients with third molar agenesis and sex (*p* > 0.05). Similarly, there was no statistically significant difference between patients with the Carabelli trait and sex (*p* > 0.05). The data are summarized in Table 1.

Among patients with third molar agenesis, 22 (29.33%) exhibited the Carabelli trait. There was no statistical significance difference between patients with third molar agenesis and Carabelli trait (*p* > 0.05). These results and *p*-values of the comparisons are shown in the Table 2.

Dental agenesis was more prevalent in the left mandibular third molars, followed by the right mandibular third molars and maxillary third molars.

The Carabelli trait was more prevalent in right maxillary first molars, followed by left maxillary first molars, right maxillary second molars, and left maxillary second molars. In the right maxillary first molars, 82 (53.59%) of the teeth did not present the Carabelli trait, 43 (28.10%) presented a negative trait, and 28 (18.30%) presented a positive trait. In the left maxillary first molars, 84 (54.54%) of the teeth did not present the Carabelli trait, 43 (27.92%) presented a negative trait, and 27 (17.53%) presented a positive trait. In the right maxillary second molars, 140 (90.90%) of the teeth did not present the Carabelli trait, 12 (7.79%) presented a negative trait, and 2 (1.29%) presented a positive trait. In the left maxillary second molars, 141 (92.76%) of the teeth did not present the Carabelli trait, 9 (5.92%) presented a negative trait, and 2 (1.31%) presented a positive trait. There was no statistical difference between molars with Carabelli traits and dental agenesis in the first and second permanent maxillary molars (*p* > 0.05). These results and the *p*-values of the comparisons are presented in Table 3.

## 4. Discussion

Dental agenesis is a highly frequent developmental dental anomaly in humans, commonly observed in clinical practice, and commonly associated with other conditions [6,7,8]. In the current study, we aimed to investigate whether Carabelli trait is associated with third molar agenesis, and although an association was not observed, some important topics and limitations should be discussed here. The main limitation of the current study is the small sample size and lack of generalizability. The lack of generalizability in a study is a limitation because it restricts the applicability of the study’s findings to broader populations or different contexts. Generalizability, also known as external validity, ensures that the results obtained from a specific sample or setting can be extended to other ethnic groups (other populations) or settings (for example, not orthodontic patients).

Third molars, although the most affected teeth by dental agenesis, are generally under-represented in clinical studies and studies focusing on the etiology of this condition. The studies commonly focus on the other type of dental agenesis [8,16,26]. This premise is justified, as third molars are the last teeth to form and erupt among all dental groups, which may contribute to their developmental fragility and common congenital absence and predisposition for other anomalies [27]. Such a fact can be cited as a contributing factor to the scarcity of studies investigating third molar agenesis and the popularization of lack of the existing scientific evidence on this topic. It is important to note that while few studies investigate third molar agenesis, it is an important subject in daily clinical practice. Third molar agenesis prevalence is influence by demographic data [8,9,10,16,26], therefore, scientific evidence suggests third molar agenesis as an important biological marker in population diversity [9,10].

Third molar agenesis has been described in association with other developmental morphological alterations in dental development [12,14,27]. Therefore, it is interesting to hypothesize a possible relationship between third molar agenesis and the Carabelli trait. The Carabelli trait is a non-metric characteristic extensively studied in dental anthropology [18,19,20]. First described in 1842 by Sir Georg Carabelli, it has been used as a critical ethnic indicator for decades, likely due to its simple observation in both living individuals and skeletal remains, thus demonstrating significant population differences in dentition [18,19,20]. The Carabelli trait was observed in significantly greater numbers in patients with the dental agenesis of lateral incisors, suggesting an association between these two developmental conditions [17]. Therefore, the aim of the present study was to hypothesize a possible association between the Carabelli trait in permanent molars and third molar agenesis. However, our results demonstrate that there is no association between the Carabelli trait and third molar agenesis in the studied population and our hypothesis was not confirmed.

The prevalence of third molar agenesis in our sample was 25.2%, and despite showing similarity to what was previously reported in the literature [9,10,16,26], our sample size appears to have been insufficient for the evaluation regarding the association with the Carabelli trait. Studies in European populations demonstrate a frequency of the Carabelli trait in nearly 90% of the population [19]; however, in our sample, the prevalence was 48.4% in permanent molars. In the study by Kerekes-Máthé et al. [17], although it does not report the prevalence of the Carabelli trait in the population, it suggests a significant sample size to ensure statistically significant association with dental agenesis.

In the present study, panoramic radiographs were used for the diagnosis of third molar agenesis. Panoramic radiographs provide a broad view of the entire mouth, including all teeth (both erupted and unerupted), jawbones, and other surrounding structures. These are especially useful for identifying missing teeth and analyzing maxilla and mandible development. It is important to note that our results differ from other scientific evidence showing a higher prevalence of third molar agenesis in females [28,29]. The prevalence of dental agenesis in other permanent teeth is also higher in females [29]. In our study, no sexual dimorphism was observed. These data corroborate with a previous study published in the literature [29].

Regarding the distribution of the Carabelli trait and sexual dimorphism, our results are like the literature, which also describes a higher frequency in males [18,19,20], although a statistical difference was not observed. In a study conducted in a Jordanian population, males showed a statistically higher prevalence of the Carabelli trait in permanent first molars [30]. In a recent study in an Indian population, these findings were reaffirmed, in which the Carabelli trait was statistically significant more prevalent in males [31,32,33]. It is suggested that studies in the German population should be replicated with a larger sample size than that employed in the present study.

Our results also demonstrate that the Carabelli trait was more frequent in first molars compared to second molars. This finding is consistent with the literature [18,19,20,30,33] and can be explained by the process of cusp formation. Enamel knots are specialized regions of the dental epithelium where the cusps starts. The enamel knots are signaling centers directing the growth of tissues surrounding it. An enamel knot need to form beyond the inhibition fields of other enamel knots, for a new cusp to form. The number and dimension of cusps depend on the spacing between enamel knots, as reflected in cusp spacing. Although the absolute spacing of cusps is similar in the first and second molars, the reduced size and more triangular form of second molars result in greater cusp spacing relative to size, and presumably, fewer opportunities for the Carabelli trait to develop [34].

When first and second molars were stratified into groups according to the classification adapted by Kamatham et al. [25], our results also did not show a statistically significant association with third molar agenesis. Third molar dental agenesis may be associated with the maxillomandibular complex variations [21,35] and affect dental dimensions [36,37], consequently impacting crown morphological development.

Our study was based on the hypothesis that these two developmental dental conditions share some genetic aspects; therefore, they would be associated. Dental agenesis and dental morphological alterations are influenced by shared genetic pathways, reflecting the intricate regulation of tooth development. Key genes such as MSX1 (Msh Homeobox 1), PAX9 (Paired Box Gene 9), RUNX2 (Runt-related Transcription Factor 2), DLX1 and 2 (Distal-less Homeobox 1 and 2) AXIN2 (Axin Antagonist 2), and EDA (Ectodysplasin A) have been implicated in both conditions due to their roles in early craniofacial and dental patterning. Mutations or genetic polymorphisms in these genes, or other genes expressed during dental development, can disrupt the signaling networks responsible for odontogenesis, leading to dental agenesis, or morphological anomalies such as peg-shaped teeth or changes in cusp patterns (such as Carabelli trait). If an association between dental agenesis and Carabelli trait was observed, these would suggest that these genetic overlaps shows that dental agenesis and the Carabelli trait are not isolated phenomena but part of a spectrum of dental developmental anomalies, providing insights into the broader genetic basis of craniofacial development. Although our study did not find an association, these could be due to the limited sample size evaluated herein. It is also possible that an association exist with other types of tooth agenesis (premolar or incisors) or that an association exists only in other populations. Therefore, future studies should explore whether similar trends are observed in other larger populations with diverse genetic backgrounds.

## 5. Conclusions

In conclusion, our study does not support the hypothesis that third molar agenesis is associated with Carabelli trait in permanent molars in the studied population. Once the sample size is a potential limitation of our study, we suggested that future studies should be performed in a larger sample.

## Figures and Tables

**Table 1:** Frequency and assessment of dental anomalies by sex.

Variable	No Third Molar Agenesis	Third Molar Agenesis	*p*-Value	No Carabelli Trait	Carabelli Trait	*p*-Value
Gender, N (%)						
Female	55 (47.41%)	19 (48.72%)	0.88	42 (51.22%)	34 (45.33%)	0.46
Male	61 (52.59%)	20 (51.28%)		40 (48.78%)	41 (54.67%)	
Total	116 (100%)	39 (100%)		82 (100%)	75 (100%)	

N = number.

**Table 2:** Evaluation of the association between third molar agenesis and Carabelli trait.

	No Carabelli Trait	Carabelli Trait	Total	*p*-Value
No third molar agenesis	65 (79.27%)	53 (70.67%)	116 (100%)	0.21
Third molar agenesis	17 (20.73%)	22 (29.33%)	39 (100%)	
Total	82 (51.6%)	75 (48.4%)	155 (100%)	

**Table 3:** Evaluation of the association between third molar agenesis and the classification of Carabelli trait according to Dahlberg [24] and Kamatham et al. [25].

	No Carabelli	Negative Carabelli	Positive Carabelli	*p*-Value
Right maxillary first molar N = 153				
No third molar agenesis	63 (76.82%)	32 (74.41%)	19 (67.85%)	0.64
Third molar agenesis	19 (23.17%)	11 (25.58%)	9 (32.14%)	
Total	82 (100%)	43 (100%)	28 (100%)	
Left maxillary first molar N = 154				
No third molar agenesis	66 (78.57%)	30 (69.76%)	19 (70.37%)	0.47
Third molar agenesis	18 (21.42%)	13 (30.23%)	8 (29.62%)	
Total	84 (100%)	43 (100%)	27 (100%)	
Right maxillary second molar N = 154				
No third molar agenesis	107(76.42%)	7 (58.33%)	1 (50%)	0.27
Third molar agenesis	33 (23.57%)	5 (41.66%)	1 (50%)	
Total	140 (100%)	12 (100%)	2 (100%)	
Left maxillary second molar N = 152				
No third molar agenesis	107(75.88%)	5 (55.55%)	1 (50%)	0.29
Third molar agenesis	34 (24.11%)	4 (44.44%)	1 (50%)	
Total	141 (100%)	9 (100%)	2 (100%)	

## Data Availability

The original contributions presented in the study are included in the article, further inquiries can be directed to the corresponding author.
